# Roles and crosstalks of macrophages in diabetic nephropathy

**DOI:** 10.3389/fimmu.2022.1015142

**Published:** 2022-11-02

**Authors:** Hai-Di Li, Yong-Ke You, Bao-Yi Shao, Wei-Feng Wu, Yi-Fan Wang, Jian-Bo Guo, Xiao-Ming Meng, Haiyong Chen

**Affiliations:** ^1^ Department of Chinese Medicine, The University of Hong Kong-Shenzhen Hospital, Shenzhen, China; ^2^ School of Chinese Medicine, The University of Hong Kong, Hong Kong, Hong Kong SAR, China; ^3^ Inflammation and Immune Mediated Diseases Laboratory of Anhui Province, The Key Laboratory of Anti-inflammatory of Immune Medicines, Ministry of Education, Anhui Institute of Innovative Drugs, School of Pharmacy, Anhui Medical University, Hefei, China; ^4^ Department of Nephrology, Shenzhen University General Hospital, Shenzhen University, Shenzhen, Guangdong, China

**Keywords:** diabetic nephropathy, macrophage, metabolic disorder, inflammation, fibrosis

## Abstract

Diabetic nephropathy (DN) is the most common chronic kidney disease. Accumulation of glucose and metabolites activates resident macrophages in kidneys. Resident macrophages play diverse roles on diabetic kidney injuries by releasing cytokines/chemokines, recruiting peripheral monocytes/macrophages, enhancing renal cell injuries (podocytes, mesangial cells, endothelial cells and tubular epithelial cells), and macrophage-myofibroblast transition. The differentiation and cross-talks of macrophages ultimately result renal inflammation and fibrosis in DN. Emerging evidence shows that targeting macrophages by suppressing macrophage activation/transition, and macrophages-cell interactions may be a promising approach to attenuate DN. In the review, we summarized the diverse roles of macrophages and the cross-talks to other cells in DN, and highlighted the therapeutic potentials by targeting macrophages.

## 1 Introduction

Diabetic nephropathy (DN) is a common microvascular complication of diabetes and a leading cause of chronic kidney disease (CKD) worldwide with high morbidity and disability (1, 2). 30%-40% of patients with diabetes develop DN, of which 5%-10% eventually progress to end-stage renal disease (ESRD). DN accounts for almost a third of the CKD caused disability-adjusted life-years globally (3).

DN is characterized by the over deposition of extracellular matrix (ECM) which leads to thickness of basement membrane, glomerular mesangial expansion and tubulointerstitial fibrosis (4, 5). Pathologically, glucose and metabolites (e.g., reactive oxygen species and advanced glycation end products) lead to podocyte loss, mesangial expansion, and endothelial injuries in glomeruli, and epithelial damage or transition in renal tubules, eventually, result cellular inflammation, glomerulosclerosis and tubulointerstitial fibrosis (5, 6). Macrophages (Mφ), as main immune cells, are involved into the pathogenesis of DN (7). High glucose and advanced glycation end products (AGEs) promote expressions of adhesion molecules, cytokines and chemokines in podocytes, mesangial cells and epithelial cells in kidney, which recruit and activate macrophages (8, 9). Activation of resident macrophages and infiltrating macrophages in diabetic kidney promote renal inflammation and fibrosis in glomeruli and tubulointerstitium (10, 11).

This review describes roles of macrophages in patients with DN as well as in animal models. We summarized the multiple roles of macrophages, including the communication between macrophages and other renal cells in DN, and the regulation of macrophages on metabolism and inflammation in DN. We also involved the new therapeutic strategies/findings targeting macrophages to prevent and treat DN.

## 2 Dynamic changes of macrophages in DN

Macrophages have dynamic changes in DN, e.g. macrophage infiltration and macrophage polarization. We summarized these findings from DN patients and animal models to preliminarily explore the roles of macrophages in the progression of DN.

### 2.1 In human

Renal biopsies from patients reveal that macrophages present in the interstitium and glomeruli at all stages of DN (11, 12). In patients with type 2 diabetes, kidney biopsy studies indicated that the number of macrophages in the glomeruli transiently increases in the stage of moderate glomerulosclerosis, but remains low at mild and advanced stages (13). The ratio of M1/M2 macrophages is dynamically changing during the progression of DN at different stages (14, 15). These macrophages traditionally classified into inflammatory M1 macrophages (CD68+/iNOS+) and M2 macrophages (CD68+/Arg-1+) (12, 14, 15). Findings from kidney biopsies showed that M1 macrophages are recruited to the kidney during the early stages of DN (Stages I and IIa). The ratio of M1 to M2 macrophages is highest at the early stages. M2 macrophages predominate at the later time point (Stage III) and the M1/M2 macrophage ratio reaches to the lowest level (14). CD163+ glomerular macrophages are positively associated with DN severity, interstitial fibrosis and tubular atrophy, and glomerulosclerosis while CD68+ interstitial macrophages are associated with decreased glomerular filtration rate and increased proteinuria (11). Studies also indicated that macrophage infiltration in tubulointerstitium is associated with the progression of renal impairment over the subsequent 5 years and macrophage accumulation in kidney is a prognostic factor for DN (11, 16). However, it is difficult to determine the dynamic changes of renal macrophages population/subpopulation due to the limited availability of human kidney biopsies in a time course of DN, especially at the early stages of DN. In addition, the onset time of the disease cannot be definitively determined and a biopsy is seldom collected at early stage as it may not be clinically appropriate (16).

### 2.2 In animals

In streptozotocin (STZ) induced type 1 diabetes mellitus (T1DM) mouse model, CD45+ and CD68+ macrophages are the most abundant leukocytes accumulating in glomerular and interstitial tissues of kidneys (17). The number of macrophages in glomeruli and tubulointerstitium is increased at 2-week after receiving STZ, and reach to a 3-fold increase at 18-week (17). Both glomerular macrophages and interstitial macrophages have been associated with hypertrophy, hypercellularity, and extracelluar matrix (ECM) deposition in glomeruli and interstitial tissues (17, 18). Consisently, macrophages accumulate in kidneys of insulin-2 Akita mutant mice, the spontaneous diabetic mice (19).

Db/db and ob/ob mice develop obesity and insulin resistance due to mutations of leptin receptors and defects of leptin production respectively. These animals are commonly used to study the pathogenesis of type 2 diabetes mellitus (T2DM) (20). In db/db mice with C57BL/6 background, glomerular and interstitial CD68+ macrophages gradually increase 10-fold at 8-month old compared to age-matched normal mice (21). These macrophages account for 90% of infiltrating leukocytes in kidneys, which are associated with blood glucose, albuminuria, and histology (21). Db/db mice with C57BLKS background at 3-month-old have a 2-fold increase of CD68+ glomerular macrophages in kidney, which are associated with the development of proteinuria (22). In an accelerated DN model by the uninephrectomy of C57BLKS db/db mice, there is an sustained increase of glomerular macrophages at 6- to 24-week old (23). In another accelerated DN model, db/db mice crossing with endothelial NOS-deficient mice, there is a 6-fold increase in glomerular CD68+ macrophages and a 5-fold increase in interstitial CD68+ macrophages at 16-week old compared with conventional db/db (24). In BTBR ob/ob mice, macrophages activation and infiltration are found in glomerulus and tubulointerstitium of kidney (25, 26). Depletion of macrophages by intraperitoneal injection of clodronate liposomes improves proteinuria and renal function in db/db mice (12).

Studies in human and animal models have shown that macrophages accumulate in diabetic kidneys and this accumulation correlates with renal injury in DN.

## 3 Origins and phenotypes of macrophages

Renal macrophages include resident macrophages and infiltrating macrophages. Renal resident macrophages include yolk sac-derived erythro-myeloid progenitors (EMPs) derived macrophages, fetal liver EMP-derived macrophages and Hematopoietic stem cells (HSCs)-derived macrophages. Some HSCs migrate into the bone marrow and spleen and are then released into the blood as circulating monocytes that can contribute to tissue-resident macrophages populations (27, 28). At the initial stage of diabetic nephropathy, resident macrophages, acting as gatekeepers to initiate or suppress immune responses, are rapidly activated by stimuli in kidney (28–30). High glucose induces high expression of adhesion molecules including intercellular adhesion molecule-1 (ICAM-1) (31) and vascular cell adhesion molecule-1 (VCAM-1) (32, 33) in vascular endothelial cells. High glucose and AGEs also promote expression of ICAM-1 in podocytes, mesangial cells and epithelial cells (34–36). They can adhere to circulating monocytes (34). ICAM-1 appears to be a key molecule promoting renal macrophage recruitment in DN (34). Meanwhile, activated resident macrophages and renal parenchymal cells such as glomerular podocytes, mesangial cells and tubular epithelial cells in DN secrete chemokines such as C-C Motif Chemokine Ligands 2 &5 (CCL2, CCL5) (37) and macrophage colony-stimulating factor 1 (CSF-1) (38), which induce circulating monocytes (Ly6C^high^ monocytes and Ly6C^low^ monocytes) to form the infiltrating macrophages in kidney and finally contribute to pathogenesis of kidney diseases (28).

**Figure 1:**
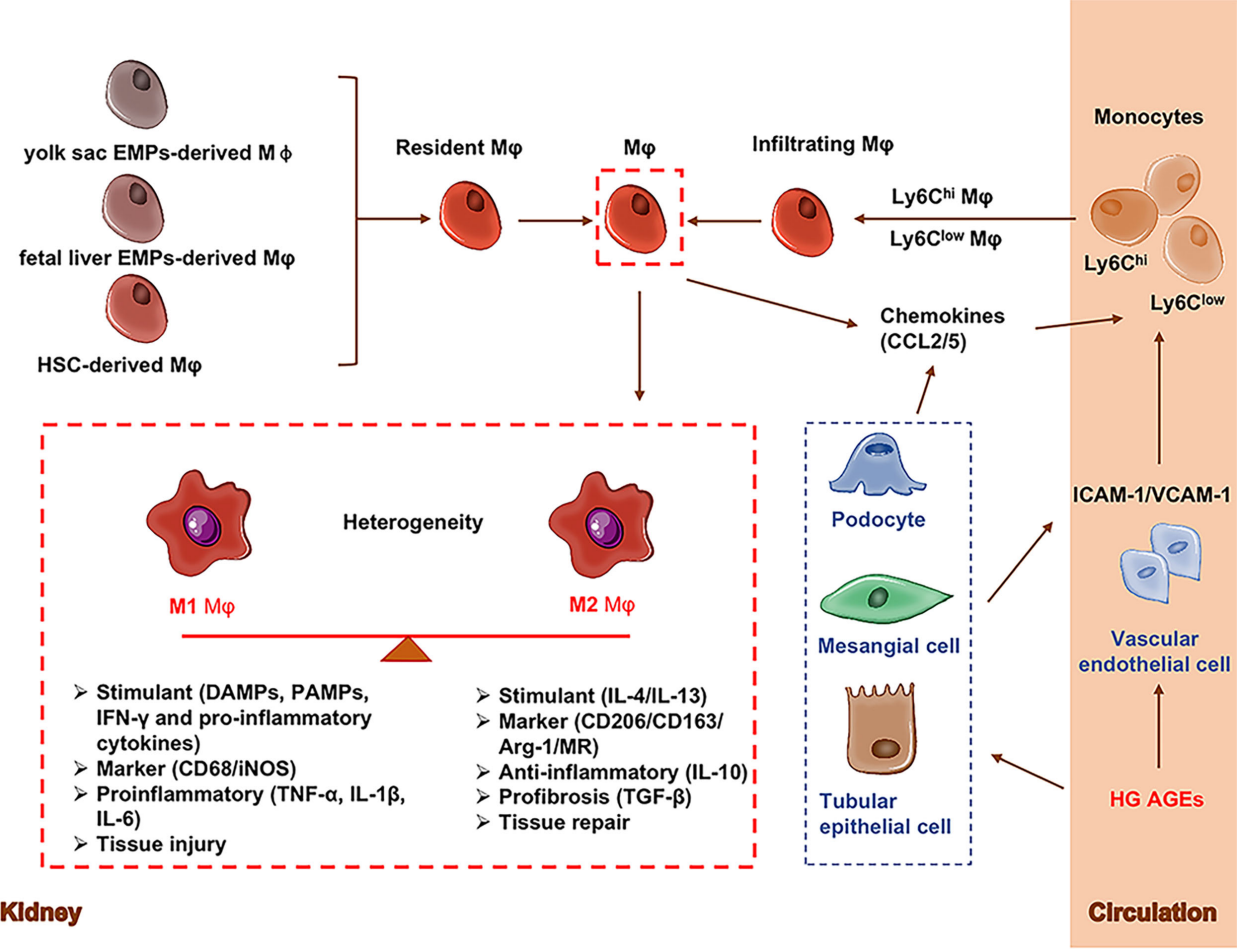
Origins and phenotypes of macrophages. Renal macrophages (Mφ) are consisting of resident macrophages and infiltrating macrophages. Resident macrophages mainly have three sources including yolk sac-derived erythro-myeloid progenitors (EMPs)-derived Mφ, fetal liver EMP-derived Mφ, and hematopoietic stem cell (HSC)-derived Mφ. During the initial stages of kidney injury, resident macrophages act as gatekeepers to initiate or suppress immune responses. Actived resigent Mφ and kidney cells release chemokines/cytokines to recruit circulating monocytes to kidney tissuse (called infiltrating macrophages). Macrophages are generally classified into classic M1 and alternative M2 macrophages. M1 macrophages (CD68/iNOS) express and secrete inflammatory cytokines (TNF-α, IL-1β, IL-6) and promote tissue inflammation. M2 macrophages (CD206/CD163/Arg-1/MR) secrete anti-inflammatory (IL-10) and pro-fibrotic cytokines (TGF-β) that promote tissue repair and fibrosis. Arg-1, Arginase-1; EMPs, Erythro-myeloid progenitors; CCL2, C-C Motif chemokine ligand 2; HSC, Hematopoietic stem cell; ICAM-1, Intercellular adhesion molecule-1; MR, Mannose receptor; Mφ, Macrophages; VCAM-1, Vascular cell adhesion molecule-1; VECs, Vascular endothelial cell.

M1 and M2 plays the opposite role in renal inflammation. At the early stage of kidney injury, macrophages are activated by pathogen-associated molecular patterns (PAMPs), danger-associated molecular patterns (DAMPs), interferon-gamma (IFN-γ) and pro-inflammatory cytokines to differentiate into proinflammatory M1 macrophages which are in response to infection or cellular damage (39). Simultaneously circulating monocytes (CD11b+Ly6C^high^) are recruited to the kidney to differentiate into pro-inflammatory M1 macrophages (40, 41). M1 macrophages have inflammatory effects with high expression of inducible nitric oxide synthase (iNOS).M1 macrophages secrete pro-inflammatory cytokines (TNF-α, IL-1β, IL-6) and promote tissue inflammation and damage (42). M2 macrophages are normally induced by interleukin 4 (IL-4) and interleukin 13 (IL-13), which suppress inflammation and promote wound repair and fibrosis (28). M2 macrophages have immunomodulatory, pro-fibrotic and repairing effects with high expression of CD206, CD163, arginase-1 (Arg-1) and mannose receptor (MR) (42). Alternative M2 macrophages secrete anti-inflammatory (IL-10) and pro-fibrotic cytokines (TGF-β) that promote tissue repair and fibrosis (42). Consistently, circulating Ly6C^low^ monocytes are recruited to the kidney to differentiate macrophages and play anti-inflammatory and pro-fibrotic roles (28). The origins and phenotypes of macrophages in the kidney are shown in **Figure 1**.

Majority of phenotype studies on DN focus on M1 and M2 macrophages. The key receptor that regulates macrophages transition from M1 to M2 is CD163. Clearance of hemoglobin:haptoglobin (Hb : Hp) complex by CD163 promotes nuclear translocation of nuclear factor erythroid 2-related factor 2 (Nrf2), increases the expression of heme oxygenase (HO)-1 and enhances release of IL-10 (43). Nrf2/HO-1, ac as therapeutic regulator, promotes the switch of M1 to M2 and improves renal function in patients (43). Hyperglycemia promotes glomerular injury by reducing the expression of renal proximal tubule AT1 receptor-associated protein (ATARP), which finally reduces accumulation of tubulointerstitial M2 macrophages in diabetic kidney (44).

The subtype classification of macrophages are more complicated than the traditional M1/M2 classification. Recent single-cell sequencing studies have shown that different macrophage populations can be more precisely described according to their gene expression patterns (45–47). Several single cell RNA-sequencing (scRNA-seq) studies have investigated the roles of macrophage subpoplulations on the pathogenesis of DN. Increased number of immune cells is shown in the glomeruli of STZ-treated mice and proved that macrophages are the predominant immune cells by assessing canonical C1qa, Cd74 and Adgre1 expression (47). The roles of different phenotype macrophages in diabetic nephropathy remain further elucidation by using more precise research methodology.

## 4 Crosstalks among macrophages and renal cells in DN

Interactions among macrophages and other renal cells (podocytes, renal tubular epithelial cells, endothelial cells, and mesangial cells) contribute to DN.

### 4.1 Macrophages and podocytes

Podocytes are important parenchymal cells of the kidney. In DN, macrophages promote podocyte injury and apoptosis. Loss of podocytes leads to proteinuria in DN. As podocytes have limited regenerative capacity, podocyte injury is an important prognostic marker for determining the severity of DN (48, 49).

In DN, high glucose (HG) activates reactive oxygen species (ROS)-p38 mitogen-activated protein kinase (MAPK) pathway in macrophages to release TNF-α and promote podocytes apoptosis (50). Macrophage depletion attenuates tubular necrosis and injury, which in turn reduces renal inflammation and fibrosis in STZ-induced diabetic rats (50). Increased T cell immunoglobulin domain and mucin domain-3 (Tim-3) activates nuclear factor κB (NF-κB)/TNF-α signaling in macrophages, which promotes podocytes injury in STZ-induced diabetic mice and db/db mice (51). Furthermore, polarization of macrophages also plays a role in podocytes. In the rat model of DN, NAD-dependent protein deacetylase sirtuin-6 (SIRT6) is decreased in macrophages under high glucose conditions. Overexpression of SIRT6 inhibits apoptosis-related genes in podocytes by activating M2 macrophages and protects podocytes from injuries *in vitro* (52). Vitamin D not only reduces macrophages infiltration and inhibits M1 macrophages activation, but also enhances M2 macrophages phenotype to prevent podocyte damage (41, 53).

In microenvironment, exosomes, as the novel communication media between cells, have been recently studied. In diabetic nephropathy, miR-21-5p in macrophages-derived exosomes promotes podocyte injury by enhancing pyroptosis through the tumor necrosis factor alpha-induced protein 3 (A20) (54). When promoting the switch of macrophages to M2 subtype, M2 macrophages can ameliorate high glucose-induced podocyte injury by inhibiting dual specificity protein phosphatase 1 (DUSP1) expression and activating autophagy by secreting exosomal miR-25-3p (55). Meanwhile, exosomes secreted by M2 macrophages attenuate LPS-induced podocyte apoptosis by regulating the miR-93-5p/toll-like receptor 4 (TLR4) axis (56).

### 4.2 Macrophages and renal tubular epithelial cells

Crosstalk between Notch and NF-κB signalings in macrophages contributes to the polarization of macrophages and the release of inflammatory cytokines/chemokines in DN. Among them, TNF-α activates renal tubular cells to undergo necroptosis. At the same time, high glucose stimulation is accompanied by the production of chemokines in renal tissue, which further increases the infiltration of macrophages (12). In addition, tubular epithelial cell-to-macrophage communication could form a negative feedback loop by extracellular vesicle (EV) to induce renal inflammation and apoptosis in DN (57). Leucine-rich α-2-glycoprotein 1(LRG1)-enriched extracellular vesicles derived from lipotoxic tubular epithelial cells activate M1 macrophages, which might be *via* a TGFβ receptor 1 (TGFβR1)-dependent manner (57). Macrophages-derived extracellular vesicles containing tumor necrosis factor-related apoptosis-inducing ligand (TRAIL) lead to renal tubular epithelial cell apoptosis in a TRAIL-Death receptor 5 (DR5)-dependent manner (57).

### 4.3 Macrophages and mesangial cells

Inflammatory cytokines released by the infiltrating macrophages promote mesangial cells to produce extracellular matrix in kidney and aggravate renal injury in DN (12). When exposed to high glucose, macrophages and mesangial cells can interact to promote the secretion of inflammatory cytokines and extracellular matrix. TGF-β activated kinase-1 (TAK1) inhibitor, 5Z-7-oxozaeenol, reduces the number of infiltrated macrophages and extracellular matrix secretion from mesangial cells when mesangial cells are co-cultured with high glucose-treated macrophages. The mechanism may be related to the inhibition of NF-κB p65 nuclear translocation, which reduces the inflammatory response and the production of extracellular matrix (58). Exosomes also function as the important communication media between macrophages and mesangial cells. Exosomes derived from high glucose-treated macrophages induce the activation and proliferation of mesangial cells, which causes mesangial expansion, and inflammatory cytokines secretion. Mechanistically, the high expression of TGF-β1 in secreted exosomes from the macrophages activates mesangial cells to produce extracellular matrix deposition *via* TGF-β1/Smad3 signaling pathway *in vivo* and *in vitro* (59).

### 4.4 Macrophages and endothelial cells

High glucose condition induces upregulation of hypoxia-inducible factor-1α (HIF-1α)/Notch1 pathway in endothelial cells, which in turn promotes the recruitment of M1 macrophages to the kidney, and mediates renal injury in db/db mice (60). The inhibition of HIF-1α/Notch1 by and peroxisome proliferator-activated receptor alpha (PPAR-α) agonist, fenofibrate, reduces M1 macrophages recruitment to prevent DN (60).

### 4.5 Macrophages-macrophages interaction

Macrophages have interactions with each other. Cytokines, chemokines and exosomes released from activated macrophages further recruit peripheral macrophages and activate macrophages. In addition, macrophage-secreted exosomes may participate the interactions. RAW 264.7 is a macrophage cell line from a tumor in a male mouse induced with the Abelson murine leukemia virus. High glucose induces exosome secretion in RAW264.7 cells (61). The study also showed that exosomes from high glucose-treated RAW264.7 cells activate macrophages *in vivo* (61).

### 4.6 Macrophage-myofibroblast transition

Recently, macrophages are identified as the major source of myofibroblasts when macrophages are stimulated with adenosine and/or TGF-β (62). The newly discovered phenomenon is firstly named as macrophage-myofibroblast transition (MMT) in 2016 (63, 64). Persistent chronic inflammation leads to progressive fibrosis of the kidneys by MMT (65). It has been proved that MMT has vital role in renal fibrosis and tumors (66, 67). Increased Adenosine level contributed to DN *via* the adenosine receptors (68). Intraglomerular monocytes/macrophages infiltration and MMT are regulated by A2B adenosine receptors (A2BAR) in STZ-induced DN rats (62). A2BAR antagonist, MRS1754, attenuates symptoms of renal function decline, glomerular fibrosis and glomerulosclerosis in DN rats by reducing intraglomerular macrophage infiltration and inhibiting MMT (62). The finding gives a hint for the treatment of diabetic nephropathy.

**Figure 2:**
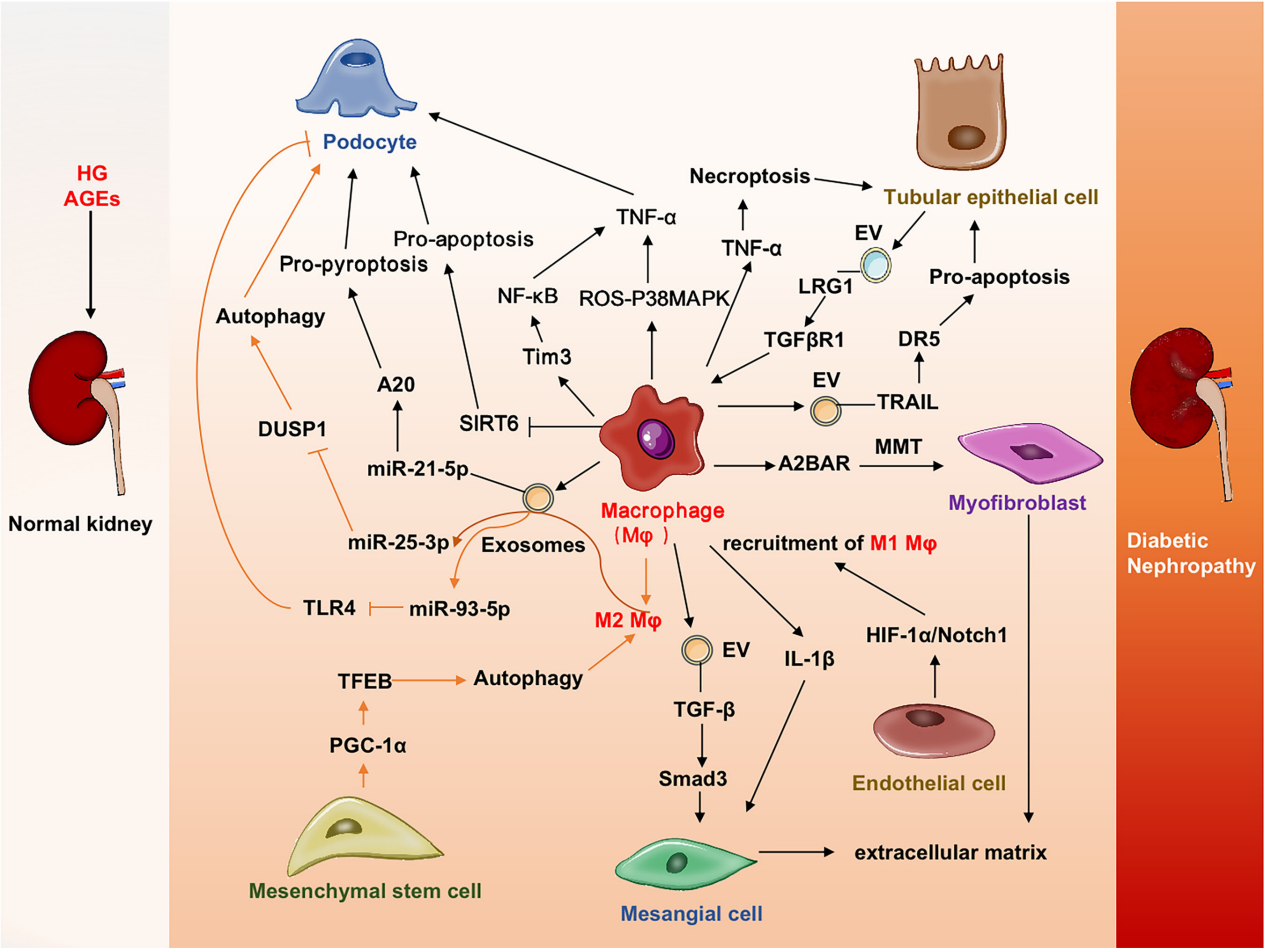
Cross-talks among macrophages and other cells in DN. Macrophages promote injuries or deaths of renal cells (podocytes, mesangial cells tubular epithelial cells, endothelial cells) by various mechanisms. Mesenchymal setem cells could promote macrophages transdifferentiated into myofibroblast and contribute to tissue fibrosis. A2BAR, A2B adenosine receptor; DR5, Death receptor 5; DN, Diabetic nephropathy;DUSP1, Dual specificity protein phosphatase 1; EV, Extracellular vesicles; HIF-1α, Hypoxia-inducible factor-1α; LRG1, Leucine-rich α-2-glycoprotein 1; MAPK, Mitogen-activated protein kinase; MMT, Macrophage-myofibroblast transition; PGC-1α, Peroxisome proliferator-activated receptor-gamma coactivator-1α; ROS, Reactive oxygen species; SIRT6, Sirtuin-6; TFEB, transcription factor EB; TGFβR1, TGFβ receptor 1; Tim3, T cell immunoglobulin domain and mucin domain-3; TLR4, Toll-like receptor 4; TRAIL, Tumor necrosis factor-related apoptosis-inducing ligand.

In conclusion, studies have shown that macrophages have multiple interactions with macrophages and renal cells to enhance renal inflammation and fibrosis (**Figure 2**). Therapy targeting macrophages could attenuate kidney injury and improve renal function in DN.

## 5 Pathogenic roles of macrophages in DN

**Figure 3:**
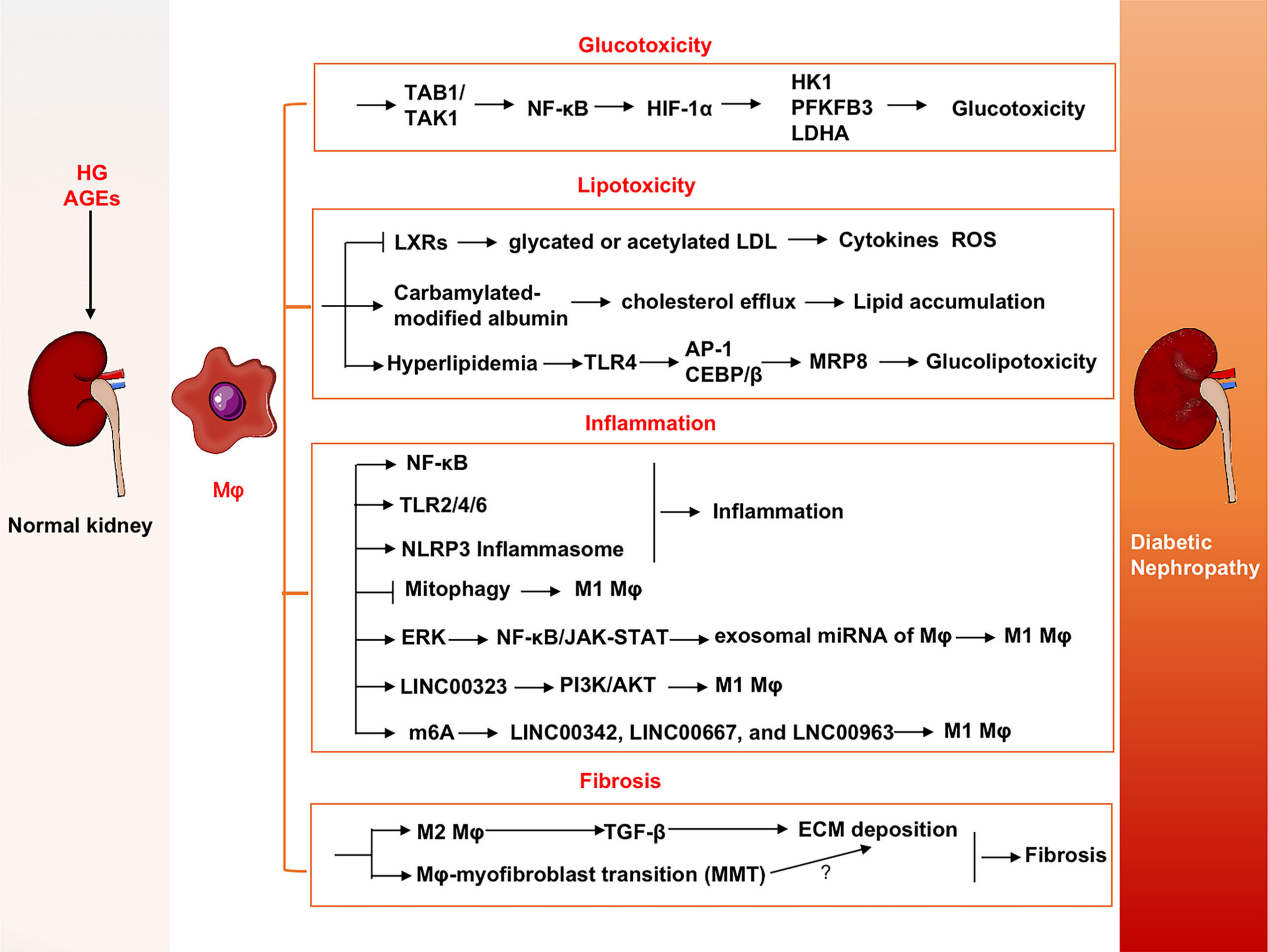
Roles of macrophages in DN progression. Macrophages contribute progression of DN *via* glucotoxicity and lipotoxicity-induced injuries, inflammation and fibrosis in kidney. DN, Diabetic nephropathy; ERK, Extracellular signal-regulated kinase; FTO, Fat mass and obesity-associated; HIF-1α, Hypoxia-inducible factor-1α; HK1, Hexokinase-1; LDHA, Lactate dehydrogenase A; LDL, Low-density lipoprotein; LncRNAs, Long noncoding RNAs; LXRs, Liver X receptors; MMT, Macrophage-myofibroblast transition; MRP8, Myeloid-associated protein 8; PDGF, Platelet-derived growth factor; PFKFB3, 6-phosphofructo-2-kinase/fructose-2, 6-biphosphatase 3; TAB1, Transforming growth factor beta-activated kinase 1-binding protein 1; TAK1, Transforming growth factor beta-activated kinase 1; TLR4, Toll-like receptor 4.

The major pathogenesis of diabetic nephropathy includes abnormal metabolic reprogramming as well as inflammation and fibrosis. Hyperglycemia and abnormal metabolites exacerbate renal damage. Roles of macrophages in specific pathological processes are summarized below and shown in **Figure 3**.

### 5.1 Metabolites-mediated macrophage reprogramming in DN

#### 5.1.1 Glucotoxicity and macrophages

In the state of diabetes, accumulation of metabolites causes damages of organs/tissues, such as kidney, liver, eyes, and nerves. During gluconeogenesis, approximately 50% of the body’s glucose is produced, of which 50% is produced in the kidneys (69). It reduces the occurrence of ketoacidosis and the risk of hyperosmolar coma but also increases the glucose load on the kidneys (70).

High glucose increases expression of glycolytic enzymes such as hexokinase-1 (HK1), 6-phosphofructo-2-kinase/fructose-2, 6-biphosphatase 3 (PFKFB3) and lactate dehydrogenase A (LDHA) which promote inflammatory responses, for example, the activation of NF-κB p65 (71). Transforming growth factor beta-activated kinase 1-binding protein 1 (TAB1) interacting with TAK1 activate NF-κB in high glucose-induced bone marrow-derived macrophages (BMDMs) and regulate macrophages activation (72, 73). Activation of NF-κB signaling pathway upregulates HIF-1α activity and promotes glycolytic metabolism (74). Downregulation of TAB1 inhibits macrophages glycolysis, polarization and inflammation through TAB1/NF-κB/HIF-1α, and further reduces albuminuria, tubulointerstitial injury, and mesangial expansion in STZ-induced DN mice (71).

#### 5.1.2 Lipotoxicity and macrophages

Under physiological conditions, lipids are responsible for maintaining intracellular metabolism, cellular communication, and membrane structural integrity. In DN, fatty acids cause cellular stress and lipotoxicity once fatty acid uptake and synthesis exceed celluar demand (75). Lipotoxicity aggravates kidney injury by activating inflammation, oxidative stress, mitochondrial dysfunction, and cell death (76). Lipid deposition in patients with DN is associated with dysregulation of lipid metabolism genes, including downregulation of fatty acid β-oxidation pathways, such as PPAR-α, carnitine palmitoyltransferase 1, acyl-CoA oxidase, and L-type fatty acid binding protein (L-FABP) (77). Lipid accumulation in renal cells promotes macrophages recruitment (78). In addition, lipotoxicity also directly activates macrophages, leading to their trans-differentiation (79). Excessive and chronic uptake of lipids by macrophages is an aggravating factor involved in glomerular injury in patients with chronic kidney disease (80).

Accumulation of lipid droplets is positively associated with renal damage in DN. Overexpression or activation of liver X receptors (LXRs) in macrophages significantly inhibits glycated or acetylated low-density lipoprotein (LDL) to induce cytokines and ROS, thereby improving renal function (81). In diabetic nephropathy, pro-inflammatory M1 macrophages participate in the development of lymphangiogenesis by stimulating vascular endothelial growth factor-C (VEGF-C) and transdifferentiate into lymphatic endothelial cells (82). Attenuated lymphoproliferation ameliorates diabetic nephropathy and high-fat diet-induced nephrolipotoxicity, suggesting a causal relationship between lipotoxicity and lymphoproliferation and a link to macrophages activation to DN (82). Carbamylated-modified serum albumin impairs macrophages cholesterol efflux in kidneys of T2DM, which results in renal damage by promoting lipid accumulation in macrophages and impairment of reverse cholesterol transport (83). Myeloid-associated protein 8 (MRP8 or S100A8) is elevated in glomeruli of T1DM and T2DM mice (84). Macrophages are the major source of MRP8 in glomeruli. Hyperlipidemia activates circulating macrophages through TLR4-mediated upregulation of MRP8, especially under hyperglycemic conditions. The synergistic effects on MRP8 production in macrophages may be mediated by fetuin A and transcription factors AP-1 and CEBP/β. Positive feedback mediated by the MRP8/TLR4 interaction in an autocrine manner enhances macrophages activation. Glomerular-intrinsic cells such as podocytes, mesangial cells, and endothelial cells can be activated through MRP8/TLR4 by resident and infiltrated macrophages. The activated cells contribute mesangial expansion and podocytes damage, further result in glomerulosclerosis, fibrosis, and proteinuria (85). Activation of MRP8/TLR4 signaling promotes macrophages-mediated glucolipotoxicity which is a novel mechanism in the pathophysiology of DN.

### 5.2 Inflammation and macrophages in DN

The release of various cytokines and chemokines from macrophages, lymphocytes and kidney cells activates inflammatory signaling pathways in macrophages which contributes to the pathogenesis and progression of DN (8, 86). Macrophages secrete a number of cytokines, including M1-related pro-inflammatory cytokines (including TNF-α, IL-1β, IL-6, IL-12, IL-15, IL-18, and IL-23), chemokines (such as MCP1/CCL2). M2 macrophages release distinct cytokine profiles including anti-inflammatory cytokines (such as IL-10), profibrotic cytokines (such as TGF-β) and chemokines (CCL17, CCL18, and CCL22) (87). Myeloid dendritic cells and CD68+ macrophages act as CCL18-producing cells (88). Among M2 macrophages-related factors, in addition to previous marker CD163, CCL18 is a recently novel cytokine and a potential biomarker to predict progression of chronic kidney disease (89). Mouse CCL8 (mCCL8) as an analog of human CCL18 shares a functional receptor with CCR8 (90). Evaluation of the M1/M2 cytokine profile suggests that renal mCCL8 expression is associated with downregulation of M1 cytokines and overexpression of M2 cytokines, thus contributing to the maintenance of chronic inflammation and renal fibrosis (87). Depletion of macrophages in DN significantly reduces the level of mCCL8 in renal tissue (12). The mechanism remains to be explored. CCL2/CC receptor 2 (CCR2) signaling plays a crucial role in the recruitment of macrophages to kidney in DN (91). In db/db mice, treatment with the CCR2 antagonist RS504393 significantly reduced infiltrating macrophages, improved insulin resistance and urinary albumin excretion and attenuated renal injury (92). Macrophages with the depletion of cyclooxygenase (COX) 2 present M1 phenotype, which enhances the infiltration of immune cells and the activation of renal macrophages, finally promotes the development of DN (93).

Multiple innate immune pathways are involved into the pathogenesis and progression of diabetic kidney disease (8, 86). Macrophages are the main phagocytic cells of the innate immune system. TLRs are activated by endogenous DAMPs during diabetes and induce tubulointerstitial inflammatory response *via* NF-κB signaling pathway (94). Tubular TLR4-high expression in renal biopsy of T2DM patients is positively correlated with interstitial macrophages infiltration and the level of HbA1c, and negatively correlated with renal function (95). Blockades of TLR2, TLR4, and TLR6 signaling by GIT27, a novel immunomodulator that targets macrophages, alleviates proteinuric effects when administered to db/db mice (96). Nod-like receptor protein (NLRP)-3-mediated inflammasome regulates inflammation *via* the cleavage of proinflammatory cytokines including pro-IL-1β and pro-IL-18 into mature forms (97). Macrophages exhibit sustained inflammasome activity in diabetic mice (98). Bruton’s tyrosine kinase (BTK) is activated in the kidneys of DN patients. Knockout of BTK attenuates macrophages−induced inflammation by inhibiting NLRP3 inflammasome activity in diabetic mice (99). Inflammatory response regulated by macrophages is related with activating NF-κB signaling pathway, which in turn increases secretion of the chemokine CCR2 (100), inflammatory cells infiltration, and M1 macrophages transformation. The β2-adrenergic receptor (β2AR) agonists including metaproterenol and terbutaline hemisulfate enhance β-arrestin2 and its interaction with IκBα, resulting in downregulation of NF-κB in macrophages of diabetic rats. β2AR agonists attenuate monocytes activation and proinflammatory and profibrotic responses in diabetic kidney and heart (101). Triggering receptor expressed on myeloid cells 1 (TREM-1) modulates macrophages phenotype towards M1 under high glucose *in vitro*, and consistently, TREM-1 expression in the renal interstitium is significantly associated with DN progression in human kidney biopsies (14).

### 5.3 Fibrosis and macrophages in DN

The continuous stimulation of high glucose or inflammatory cytokines/chemokines that primarily promoting tissue repair results excessive deposition of extracellular matrix and renal fibrosis (102). Accumulation of macrophages in diabetic kidneys was strongly correlated with interstitial myofibroblast accumulation and interstitial fibrosis (10, 37, 103). MMT derived myofibroblast may play major role on renal fibrosis. MMT cells have predominat M2 phenotype in both human and mouse kidney fibrosis (63, 64). M1 to M2 polarization is a key mechanism contributing to renal fibrosis in DN. M1 macrophages promote inflammation and injury, and M2 macrophages are anti-inflammatory and promote fibrosis by producing IL-10, TGF-β, etc. (104).

### 5.4 Mitophagy and macrophages in DN

High glucose plays an important role in macrophage adhesion and migration by regulating autophagic activity in diabetic nephropathy (105). Mitophagy is downregulated in macrophages in STZ-induced diabetic rats. Macrophages treated with mitochondrial inhibitor (3-MA) impair mitophagy and tend to switch to M1 phenotype (iNOS+ and TNF-α+), but macrophages treated by the mitochondrial activator (rapamycin) have less high glucose-induced M1 transition and more M2 phenotype (MR+ and Arg-1+) (106). These results suggest that mitochondria-regulated autophagy is involved in the regulation of M1/M2 macrophages switch in diabetic nephropathy.

### 5.5 Epigenetic modification of macrophages in DN

Epigenetics include DNA methylation, RNA methylation, and non-coding RNAs, etc, affecting the stability and expression of target genes, and then regulating related pathways and disease progression (86). In DN, ERK regulates M1 macrophage activation and alters exosomal miRNA expression (including miR-193a-3p, miR-1260b and miR-3175) of the macrophage through the NF-κB/JAK-STAT pathway (107). Increased long noncoding RNA LINC00323 promotes M1 macrophage polarization through PI3K/AKT signaling in DN (108). Recently, RNA methylation, especially m6A, has been found to contribute to the pathogenesis of DN. The m6A methylation modification plays a crucial role in the occurrence and development of metabolic diseases such as obesity and T2DM by regulating glucose and lipid metabolism and immune inflammation (109). Analysis of m6A-modified lncRNA expression in DN reveales that M1 macrophage polarization-related lncRNAs (LINC00342, LINC00667, and LNC00963) are indirectly associated with the downstream demethylase fat mass and obesity-associated (FTO) during m6A methylation recognition and transfer. Meanwhile, m6A and RNA binding motif protein 15 (RBM15) are involved in the immune regulation of M1 macrophages, and there may be a potential interaction between RBM15 and WTAP to regulate lncRNA methylation in M1 macrophages. It suggests that m6A methylation transfer enzymes RBM15 and WTAP and m6A demethylase FTO affect M1 macrophages polarization and lncRNA methylation in M1 macrophages in DN (110).

## 6 Therapeutic potentials of macrophages in DN

Current clinical treatments for diabetic nephropathy are to control symptoms (e.g., hyperglycemia, hyperlipidemia, and hypertension). These regimens include the use of insulin or insulin sensitizers (eg, PPAR-agonists), insulin release stimulators (eg, gliclazide), cholesterol-lowering statins, and inhibitors of the renin-angiotensin-aldosterone system (RAAS) (111). These therapies have direct effects on macrophages activity and indirectly inhibit renal macrophages recruitment to slow the development and progression of DN.

Immune regulation plays indisputable roles in the development and progression of DN, such as infiltration of immune cells, release of proinflammatory cytokines and chemokines, and formation of immune complexes in kidney (112, 113). The blockade of macrophages infiltration as well as the inhibition of MCP-1 and CCR2 have beneficial effects in clinical trials and experimental models of DN (37, 114). The suppression of M1 polarization or induction of M2 polarization could reduce diabetic kidney injury (115). The inhibition of wasted exosomes or metabolites from macrophages could also improve renal function (77)

Several compounds have been evaluated for treating diabetic nephropathy pre-clinically. Sarpogrelate hydrochloride, a 5-hydroxytryptamine (5-HT2A) receptor antagonist, alleviates DN in db/db mice by inhibiting macrophage activity and related inflammatory responses (116). Fasudil, a potent Rho-kinase inhibitor, ameliorates DN both in db/db mice and rat DKD model by the induction of M2 macrophage polarization and the reduction of M1 macrophage polarization (117). 

Studies also show that natural compounds have therapeutic potential to regulate macrophage and beneficial for DN. Loganin, an iridoid monoterpenoid, ameliorates kidney injury by inhibiting macrophages infiltration and activation through MCP-1/CCR2 signaling pathway in DN (118). Schisandrin C, a dibenzocyclooctadiene lignan from Schisandra chinensis (Turcz.) Baill, protects against DN by promoting M1 to M2 polarization of macrophages *via* the polarization-dependent Swiprosin-1/IFN-γ-Rβ signaling pathway (119). Paeoniflorin, a monoterpene glycoside from Paeonia lactiflora, prevents macrophages activation by inhibiting TLR2/4 signaling in diabetic kidney (120). Hypericin, a naphthodianthrone from Hypericum (Saint John’s wort), or quercetin-3-O-galactoside ameliorates mouse DN by promoting the polarization of macrophages from M1 to M2 phenotype and the differentiation of CD4+ T cells into Th2 and Treg populations (121).

Preclinical studies have shown the promising effects that targeting macrophages could attenuate diabetic kidney injury. However, the effects have to be warranted by rigorous clinical trials.

In addition, mesenchymal stem cells (MSCs) treatment has been shown to improve DN by reducing proteinuria and attenuating glomerular damage *in vivo*. Early intervention of MSCs protects against renal injury by restoring homeostasis of immune microenvironment and preventing renal dysfunction and glomerulosclerosis (122). In DN mice, MSCs could mediate the activation of transcription factor EB (TFEB) *via* peroxisome proliferator-activated receptor-gamma coactivator-1α (PGC-1α) pathway, and subsequently restore lysosomal function and autophagy activity in macrophages, which induces anti-inflammatory M2 macrophage phenotype and ameliorates renal injury (123, 124). Meanwhile, MMT is newly identified phenomena in several diseases and inhibition of MMT is potential to suppress the fibrosis (62, 65). It remains unclear whether MMT contributes to DN. Macrophages stabilized by neutrophil gelatinase-associated lipocalin (NGAL) retain them in M2 phenotype which increases anti-inflammatory IL-10 secretion and attenuates podocyte loss (115). The clinical effect of NGAL on DN remains unexplored. Cell therapy targeting macrophages is also a potential trend in the treatment of DN which needs to be further determined by clinical studies.

## 7 Summary

Human and animal studies suggest that macrophages play multiple roles in the development and progression of diabetic nephropathy (20, 125). Metabolic abnormalities (76, 77) and immune-inflammatory responses (10) are key links in the pathogenesis of DN. Both renal inflammation and systemic inflammation in DN are attributed to macrophages, and their mutual influence is indistinguishable, which needs to be explored in the future. In DN, DAMPs, PAMPs, lipotoxic or glucotoxic signals from the microenvironment of diabetic kidney trigger macrophages activation or polarization, leading to the release of inflammatory cytokines and chemokines, and intracellular metabolic reprogramming in kidney (38). The released cytokines or exosomes promote communication between macrophages and renal cells (including podocytes, mesangial cells, endothelial cells), and further accelerate the injury of these cells. Meanwhile, chemokines and inflammatory cytokines enhance recruitment of monocytes into kidney and differentiation into infiltrating macrophages (37). In addition, metabolic reprogramming in macrophages disturbs glycolysis and lipid synthesis, resulting in renal inflammation and fibrosis, and even glomerulosclerosis in DN.

Past studies have demonstrated the multiple roles and strong plasticity of macrophages in DN. The treatments using different strategies of targeting macrophages could suppress activation of macrophages by reducing inflammation and metabolite wastes, and regulate intercellular communication, finally attenuate diabetic kidney injury. Findings from these studies have therapeutic potential and need to be warranted by clinical trials.

## Author contributions

HC and X-MM conceived, revised and edited the manuscript. H-DL and Y-KY collected studies and drafted the manuscript. B-YS and W-FW revised the manuscript. Y-FW and J-BG assisted in data extraction. All authors approved the final version of the manuscript for publication.

## Funding

The study was supported by Shenzhen Science & Innovation Fund (JCYJ20180306173745092, JCYJ20210324114604013); HKSAR General Research Fund (17109019, 17113416); Seed Fund for Basic Research (202011159210, 202111159235); Research Start-up Foundation of Shenzhen University (SZU) and Natural Science Foundation of Shenzhen University General Hospital (SUGH2020QD011, SUGH2020QD021), and Science Technology and Innovation Committee of Shenzhen Municipality (JCYJ20210324094804013).

## Conflict of interest

The authors declare that the research was conducted in the absence of any commercial or financial relationships that could be construed as a potential conflict of interest.

## Publisher’s note

All claims expressed in this article are solely those of the authors and do not necessarily represent those of their affiliated organizations, or those of the publisher, the editors and the reviewers. Any product that may be evaluated in this article, or claim that may be made by its manufacturer, is not guaranteed or endorsed by the publisher.
